# Decision Support Tools for Regenerative Medicine: Systematic Review

**DOI:** 10.2196/12448

**Published:** 2018-12-19

**Authors:** Ching Lam, Edward Meinert, Abrar Alturkistani, Alison R Carter, Jeffrey Karp, Aidong Yang, David Brindley, Zhanfeng Cui

**Affiliations:** 1 Department of Engineering Science Institute of Biomedical Engineering University of Oxford Oxford United Kingdom; 2 Healthcare Translation Research Group Department of Paediatrics University of Oxford Oxford United Kingdom; 3 Global Digital Health Unit Department of Primary Care and Public Health Imperial College London London United Kingdom; 4 Division of Engineering in Medicine Department of Medicine Brigham and Women’s Hospital Boston, MA United States

**Keywords:** decisional tool, systematic review, regenerative medicine, cell therapy, decision support techniques, cell- and tissue-based therapy

## Abstract

**Background:**

Decisional tools have demonstrated their importance in informing manufacturing and commercial decisions in the monoclonal antibody domain. Recent approved therapies in regenerative medicine have shown great clinical benefits to patients.

**Objective:**

The objective of this review was to investigate what decisional tools are available and what issues and gaps have been raised for their use in regenerative medicine.

**Methods:**

We systematically searched MEDLINE to identify articles on decision support tools relevant to tissue engineering, and cell and gene therapy, with the aim of identifying gaps for future decisional tool development. We included published studies in English including a description of decisional tools in regenerative medicines. We extracted data using a predesigned Excel table and assessed the data both quantitatively and qualitatively.

**Results:**

We identified 9 articles addressing key decisions in manufacturing and product development challenges in cell therapies. The decision objectives, parameters, assumptions, and solution methods were analyzed in detail. We found that all decisional tools focused on cell therapies, and 6 of the 9 reviews focused on allogeneic cell therapy products. We identified no available tools on tissue-engineering and gene therapy products. These studies addressed key decisions in manufacturing and product development challenges in cell therapies, such as choice of technology, through modeling.

**Conclusions:**

Our review identified a limited number of decisional tools. While the monoclonal antibodies and biologics decisional tool domain has been well developed and has shown great importance in driving more cost-effective manufacturing processes and better investment decisions, there is a lot to be learned in the regenerative medicine domain. There is ample space for expansion, especially with regard to autologous cell therapies, tissue engineering, and gene therapies. To consider the problem more comprehensively, the full needle-to-needle process should be modeled and evaluated.

## Introduction

### Rationale

Decisional tools or decision support tools are tools that can be used to support complex decision making and problem solving. Since their advent in the 1970s [1], these tools have been used to support evidence-based decision making in various industries, including health care [2], agriculture [3], and the environment [4].

In the biopharmaceutical industry, decisional tools have been applied to monoclonal antibody and vaccine manufacturing decisions for over 20 years. These tools have proved to be useful for understanding cost structures and risks in order to inform decisions in various areas, including technology evaluation, facility fit, and capacity planning [5-10].

Decision support tools such as cost-of-goods modeling have proved themselves instrumental in informing the industry about the economic drivers in switching to new technologies. One such example is the shift from stainless steel to single-use production strategies for biologics over the last 15 years across the biopharma industry, which allowed faster campaign turnover, lower initial capital costs, and manufacturing cost savings [7,9,11]. Through providing a better understanding of the cost drivers for change, decisional tools were able to help build a valid commercial case to influence decision makers in making important business and bioprocess decisions, from technology choice and process change, to supply chain and project portfolio management [6,12-15].

Regenerative medicine, as defined by Mason and Dunnill in 2008, “replaces or regenerates human cells, tissues or organs, to restore or establish normal function, with approaches such as use of soluble molecules, gene therapy, stem cell transplantation, gene therapy, tissue engineering and the reprogramming of cell and tissue types” [16]. By 2018, the field had seen major breakthroughs. With US Food and Drug Administration approvals of the genetically modified T-cell therapies tisagenlecleucel (Kymriah) and axicabtagene ciloleucel (Yescarta) for refractory or relapsed acute lymphoblastic leukemia and large B-cell lymphoma, respectively, and voretigene neparvovec (Luxturna) for retinal dystrophy [17], the industry is slowly living up to its expectations. As more regenerative medicine products are commercialized, decisions such as cost-of-goods optimization and process design become more critical.

### Objectives

Rekhi et al [18] reviewed the existing decisional tools for monoclonal antibodies and cell therapy bioprocessing and identified plenty of room for expansion. With this review, we aimed to provide a systematic update of the regenerative medicine decision support tool landscape, with a focus on tissue engineering, and cell and gene therapies, to identify the gaps in the literature and inform future development of decisional tools in the area.

The key research questions this review aimed to address were as follows. First, what decisional tools are available in the regenerative medicine domain? Second, what issues have been addressed? Third, what are the gaps in decisional tools for regenerative medicine?

## Methods

We conducted this systematic review following the Preferred Reporting Items for Systematic Reviews and Meta-Analyses (PRISMA) guidelines. As we used only publicly available information, the review did not require ethics review board approval.

### Eligibility Criteria

To be included in this review, each article had to meet the following criteria: (1) addressed regenerative medicines, either autologous or allogeneic, (2) described a decisional tool, and (3) was available in English.

We enforced the following exclusion criteria to allow the review to focus on the outcomes of fresh research reported with sufficient details: (1) review articles, (2) conference abstracts, and (3) book chapters.

### Search Strategy and Study Selection

We searched the literature on May 23, 2018 to identify suitable studies indexed in the MEDLINE database and electronically identified the bibliographical references of the articles. We also manually searched Google Scholar.

To find relevant studies, we used the keywords in Textbox 1.

We constructed a search string by pairing a regenerative medicine term and a decisional tool search term—for example: (regenerative medicine) AND bioprocess economics; (cell therapy) AND bioprocess design.

We screened all titles and abstracts that we identified for relevance. Subsequently, we obtained full-text articles and reviewed eligible articles.

### Data Collection

**Figure 1:**
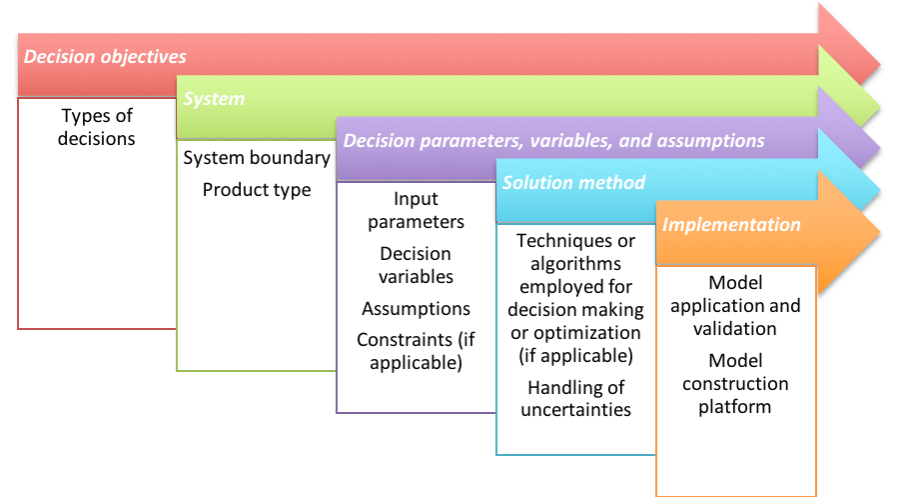
Key data extracted from the eligible literature.

We analyzed the relevant articles in five aspects that are typically shared by decision support tools found in the literature (Figure 1). We extracted key data (Figure 1) from each source, by following the same structure, into a predesigned Excel spreadsheet (Microsoft Corporation).

**Search terms.**

Regenerative medicine search items

- Regenerative medicine
- Cell therapy
- Tissue engineering
- Gene therapy
- Exosomes

Decisional tools search terms

- Bioprocess economics
- Bioprocess design
- Decision* tool
- Evaluation framework

## Results

**Figure 2:**
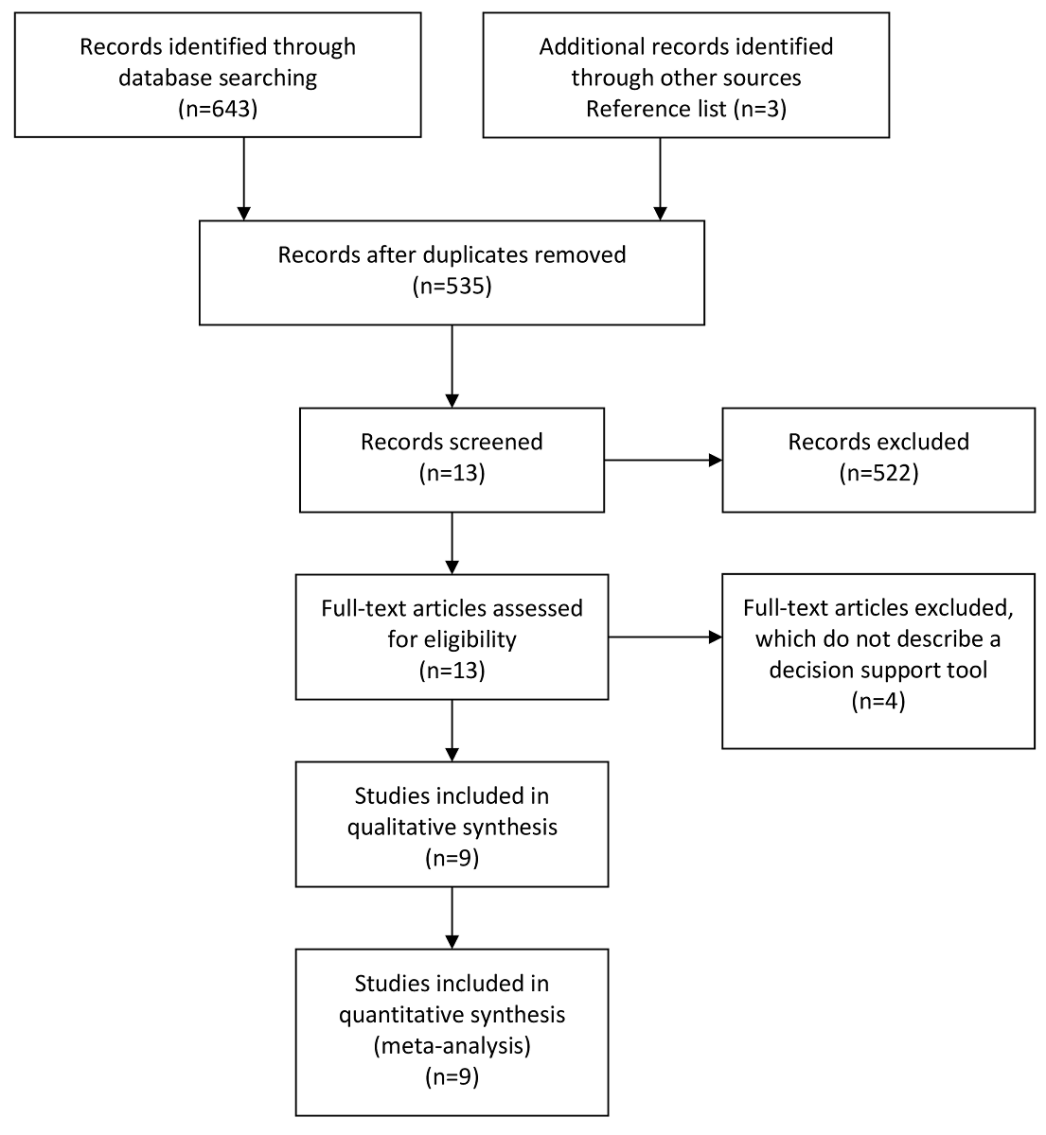
Preferred Reporting Items for Systematic Reviews and Meta-Analyses(PRISMA) flowchart of the literature review process.

Figure 2 shows the PRISMA flowchart of the literature search process. The database searches identified 646 articles for review. At the screening stage, we deemed 13 articles to be relevant for full-text eligibility assessment. We excluded 4 full-text articles after screening, as they did not contain a description of a decisional tool and, hence, did not meet the eligibility criteria. Thus, we identified 9 articles that met the inclusion criteria and reviewed them in full detail for subsequent assessments. Multimedia Appendix 1 shows the executed data abstraction form. The small number of articles we initially identified and the small number resulting after screening indicate the novelty of this research area.

### Decision Objectives

Table 1 [19-27] shows the various decision objectives of published decisional models. Ungrin et al aimed to enhance the upstream cell expansion yield [19]. Lambrechts et al [20] described a visualization tool for upstream expansion processes cited from other literature, and hence did not have a decision objective. All other models focused on optimization of the costs for manufacturing or product development. Manufacturing cost of goods was further broken down to show subcategories such as raw material, labor, consumables, and capital equipment in various analyses. Product development costs relate to investment required to bring the product from bench to bedside, including particularly clinical trial costs. Optimizing these costs is critical in the sustainable development of companies and their operational efficiency. Project net present value (NPV) is a commonly used method in project evaluation [28]. Through evaluating the NPV as an impact of process change in the development timeline, Hassan et al [21] reflected the risks and benefits of making a process change from one technology to another.

**Table 1:** Decision objectives of various decisional models in the reviewed articles.

Decision objectives	Articles
Operational yield for cell expansion process	Ungrin, 2012 [19]
Cost of goods	Upstream: Simaria, 2014 [22] Downstream: Hassan, 2015 [23] Overall: Weil, 2017 [24]; Harrison, 2018 [25]; Jenkins, 2018 [26]
Investment costs	McCall, 2013 [27]; Hassan, 2016 [21]
Risk-adjusted net present value	Hassan, 2016 [21]
Not applicable	Lambrechts, 2016 [20]

### System

#### System Boundary

Parnell et al [29] define a system boundary as a physical or conceptual boundary that contains all the essential elements, subsystems, and interactions necessary to address a systems decision problem. Different decision objectives would motivate different definitions of systems boundaries.

We generalized the systems in the eligible articles into two types: (1) product development systems, and (2) manufacturing and supply chain systems.

##### Product Development Systems

Two identified articles looked into the development phase of cell therapies, both describing generic processes that can be applied to any cell therapies. McCall [27] defined the systems boundary as being between preclinical trials and phase 3 clinical trials in order to look into the costs of developing a cell therapy. Hassan et al [21] defined their systems boundaries as being between phase 1 clinical trials and regulatory approval in order to study the impact of process changes along the development phases on NPV of their project.

##### Manufacturing and Supply Chain Systems

**Figure 3:**
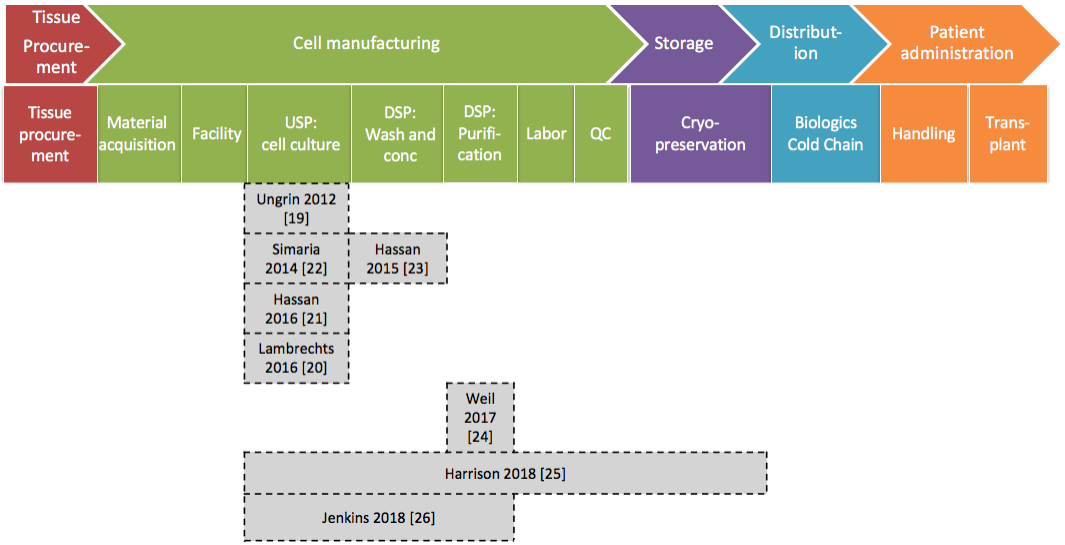
Coverage of existing decisional tools. conc: concentration; DSP: downstream processing; QC: quality control; USP: upstream processing.

A total of 8 articles investigated decision making for manufacturing and supply chain systems. Figure 3 shows the system boundaries in these 8 articles mapped against the needle-to-needle or patient-to-patient (ie, from patient tissue procurement to therapy administration) cost-of-goods roadmap proposed by Lipsitz et al [30].

Ungrin et al [19] and Lambrechts et al [20] addressed optimization of the cell expansion process upstream through experiments, bioprocess modeling, and visualization. Hassan et al [21] focused on process change impacts along the product development pathway using the change of upstream processing technology as an illustrative example; hence we have included their study in this section as well.

Simaria et al [22], Hassan et al [23], Weil et al [24], Harrison et al [25], and Jenkins and Farid [26] evaluated different technology options for the studied steps within their defined system boundaries to better understand the advantages, disadvantages, and bottlenecks in adopting different technology options and their implications for manufacturing cost of goods.

#### Product Type

All the articles we identified focused on cell and ex vivo gene therapy; we found no decisional tools in tissue engineering or in vivo gene therapy. Table 2 [19-27] shows the product types and type of transplant considered in the articles.

There was considerably more focus on allogeneic therapies and mesenchymal stem cells (MSCs) as the cell source. The study by Weil et al [24] is the only one in this review that considered autologous processes, but it focused only on downstream affinity purification.

### Decision Variables, Parameters, and Assumptions

Decision variables conceptually or mathematically represent decisions to be made in order to (best) achieve the decision objectives. For a given decision objective, the determination of a decision variable is typically affected by either internal or external characteristics, or by both of them, which are referred to as decision parameters. In other words, decision parameters, together with other assumptions taken for internal or external settings, form the input to a decision process, while the determined values for the decision variables are its output.

#### Decision Variables

Decision variables are variables that the decision-maker controls. Such variables are dependent on the decision objectives and the problems they seek to answer.

For product development systems, the models seek to answer to objectives such as minimizing development duration, risks, and investment costs. The timing for technology change was the decision variable modeled by Hassan et al to study the impact of process change in product development on the project NPV [21]. McCall [27] studied the impacts of product development risks and uncertainties, as well as rework probability on the investment costs; as there was no optimization module in this study, no decision variable was identified.

**Table 2:** Cell types and type of transplant.

Transplant type	Cell type				
	Mesenchymal stem cells	Chimeric antigen receptor T-cell	Human pluripotent stem cell/induced pluripotent stem cells	Not specified	Not applicable
Allogeneic	Hassan, 2015 [23], Hassan, 2016 [21], Lambrechts, 2016 [20], Harrison, 2018 [25]	Jenkins, 2018 [26]	N/A^a^	Simaria, 2014 [22]	N/A
Autologous	N/A	N/A	Weil, 2017 [24]	N/A	N/A
Not specified	N/A	N/A	Ungrin, 2012 [19]	N/A	N/A
Not applicable	N/A	N/A	N/A	N/A	McCall, 2013 [27]

^a^N/A: not applicable.

**Figure 4:**
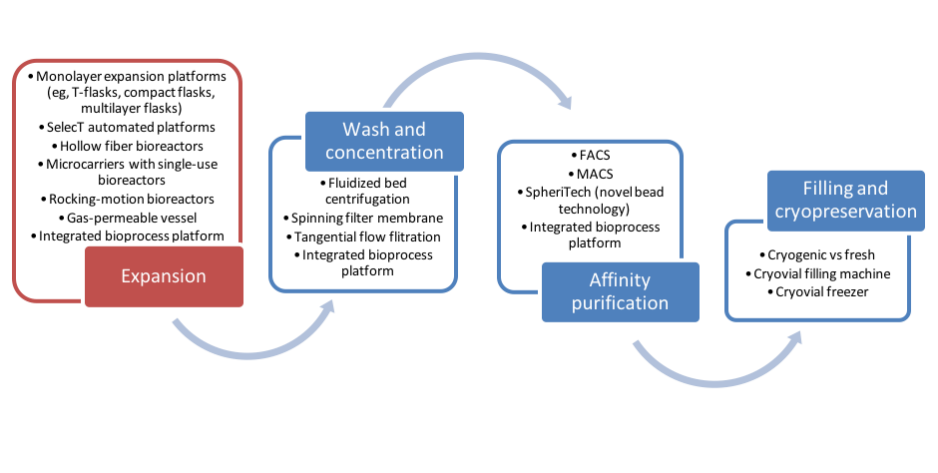
Upstream and downstream operations considered in the reviewed articles. FACS: fluorescence-activated cell sorting; MACS: magnetic-activated cell sorting.

For manufacturing and supply chain systems, the models seek to answer to objectives such as process yield [19] and manufacturing cost minimization [22,24-26]. In these systems, the choice of unit operations in the manufacturing process sequence, chosen equipment, and their capacities are critical decision variables to be considered. Figure 4 shows the upstream and downstream unit operations options considered in the articles. Together with process parameters, process flowsheet, and requirements, the processes were modeled in order to understand the associated cost of goods. Conclusions on the relative advantages of various technology options under different demand scenarios were drawn for bottleneck analysis and decision recommendations.

#### Input Parameters

Input parameters are defined as “a constant element or factor, especially serving as a limit or boundary” [31]. These are inputs defined as prerequisites of the objective function; in other words, they are constants of the objective function and not the values to be optimized. In the 9 articles we identified, these included scale, throughput, demand goal, and technical process parameters.

#### Scale, Throughput, and Demand Goal

Early articles in the area generally looked at demands several times more than the recent articles. As more commercial case studies of regenerative medicine products arise, the demand landscape is better understood and the estimation for cost-of-goods modeling has been lowered from the monoclonal antibodies ballpark (1000-500,000 doses of allogeneic MSCs per year [22] to around 2500 doses/y for a regional center for allogeneic MSCs [25] and 500-5000 doses/y for chimeric antigen receptor T cells (CAR-T) [26]). Demand scenarios were chosen depending on the cell type and the therapeutic target. As more and more real-world commercial cases have emerged, recent articles gave a lot more consideration to the clinical applications and their specific demands and, hence, proposed more realistic demand scenarios.

#### Upstream and Downstream Operations Process Parameters

For upstream operations, comparisons were drawn from a range of equipment scale, cell culture modes, and extent of automation. Table 3 [19,22,25,26] summarizes process parameters previously considered and explicitly mentioned in their respective articles.

In all the articles, planar culture flasks (eg, T-flasks and multilayer flasks) were consistently found to be the most expensive of all evaluated technologies for allogeneic therapies and infeasible for higher cell number per lot. The number of cells harvested per surface area was found to be the most important cost driver, as it dictates the number of expansion units required and, hence, the raw materials and consumables requirements [22,26].

Table 4 [23,24,26] shows the process parameters for downstream processing discussed in the articles.

**Table 3:** Input process parameters for upstream processing.

Input process parameters		Ungrin, 2012 [19]	Simaria, 2014 [22]	Harrison, 2018 [25]	Jenkins, 2018 [26]
Studied technologies		Microwell	T-flasks, multilayers, compact flasks, compact multilayers, multilayer bioreactors, hollow fiber bioreactors	T-175 flasks, SelecT automated platform	Planar culture flasks, rocking-motion bioreactor, gas-permeable vessel, integrated bioprocess platform
**Cell culture process parameters**					
	Population doublings	Yes	No	No	No
	Inoculation cell count	No	No	Yes	No
	Seeding density	No	Yes	Yes	No
	Harvest density	No	Yes	No	Yes
**Technology process parameters**					
	Surface area	Yes	Yes	No	No
	Equipment size and volume range	No	Yes	Yes	Yes
	Number of expansion stages	No	Yes	No	No
	Perfusion rate	No	No	No	Yes
	Maximum units	No	Yes	No	No
	Biosafety cabinet requirement	No	Yes	No	No
	Incubator capacity requirement	No	Yes	Yes	No
**Time duration assumptions**					
	Seed time	No	Yes	No	No
	Feed time	No	Yes	No	No
	Harvest time	No	Yes	No	No
	Cell culture duration	No	No	Yes	No
**Material use and cost assumptions**					
	Media requirements	No	Yes	Yes	Yes
	Labor requirements	No	Yes	Yes	Yes
	Consumable unit price	No	Yes	Yes	Yes
	Capital charge	No	Yes	Yes	Yes

**Table 4:** Input process parameters for downstream processing.

Input process parameters		Hassan, 2015 [23]	Weil, 2017 [24]	Jenkins, 2018 [26]
Wash and concentration: studied technologies		Tangential flow filtration, fluidized bed centrifugation	N/Aa	Fluidized bed centrifugation, spinning filter membrane, integrated bioprocess platform
Purification: studied technologies		N/A	Fluorescence-activated cell sorting, magnetic-activated cell sorting, novel bead	Magnetic-activated cell sorting, integrated bioprocess platform
**Technology process parameters**				
	Number of washes/cycles	Yes	Yes	No
	Equipment size and volume range	Yes	Yes	Yes
	Maximum cell processing capacity	Yes	Yes	Yes
	Step yield	Yes	Yes	Yes
**Time duration assumptions**				
	Maximum time	Yes	No	No
**Material use and cost assumptions**				
	Raw material requirements	Yes	Yes	Yes
	Labor requirements	Yes	Yes	Yes
	Consumable unit price	Yes	Yes	Yes
	Capital charge	Yes	Yes	Yes

^a^N/A: not applicable.

The downstream process starts with the wash and concentration step, and common wash concentration unit operations were discussed in detail by Hassan et al [23] and Jenkins and Farid [26]. Hassan et al reported that wash and concentration downstream steps were a bottleneck for high-cell-dose lots at high demand. As the demand estimate was lowered in the study of Jenkins and Farid, wash and concentration was no longer shown to be a bottleneck except in integrated bioprocess platforms such as CliniMACS Prodigy, which has a relatively smaller volume-reduction capacity.

Following wash and concentration, affinity purification has also been a target for modeling and optimization. Weil et al [24] and Jenkins and Farid [26] looked into affinity purification for autologous induced pluripotent stem cells and allogeneic CAR-T cells, respectively. Weil et al compared fluorescence-activated cell sorting (FACS) versus magnetic-activated cell sorting (MACS) and evaluated a novel technology that does not require cell labeling. MACS was determined to be more cost effective for dose sizes with a higher cell count (>7.0×10^7^ cells/dose), as FACS is limited by its process scale. The model by Jenkins and Farid [26], for affinity purification, considered only MACS and integrated bioprocess platform.

#### Assumptions and Constraints

The articles made other assumptions besides technology-related assumptions and constraints.

For scheduling-related problems where one task follows another, task precedence constraint was used. McCall [27] used the task precedence constraint for dictating the start and end of a task, which is useful for setting up the scheduling problem. Iteration loops were built in the development pathway with assumption of learning. Hassan et al [21] assumed a linear project development pathway with failure probability. They constructed a database with information on clinical trial development times and failure rates of all 592 commercial cell therapy projects from 1981 to 2011 to estimate the duration and failure rate of similar products. This approach allows more industrially relevant benchmark assumptions to be made and, hence, gives rise to higher-quality results.

For resource utilization, McCall [27] assumed a fixed and steady-rate consumption of resources and a renewable resource pool throughout the project duration. Similar assumptions were made in all the other cost models to better understand the impact of resource utilization on the overall cost of goods. For instance, Simaria et al [22] showed that efficient use of equipment and facility can lower the depreciation costs shared across doses. Harrison et al [25] looked into the impact of human resource turnover in detail to understand the impact of increased operators on the relative cost of labor in overall cost of goods.

Having reasonable cost assumptions is one of the most important factors determining the validity of the model. Table 5 [22-26] shows some of the cost assumptions used in various models.

**Table 5:** Extracted quality control (QC), labor, and facility cost assumptions.

Cost type	Simaria, 2014 [22]	Hassan, 2015 [23]	Weil, 2017 [24]	Harrison, 2018 [25]	Jenkins, 2018 [26]
QC	US$10,000/lot	US$10,000/lot	QC costs per dose = £3250	QC costs based on Athersys Simpler assay panel: US$5934.53 Advanced assay panel: US$37512	QC quality assurance cost = 1 × operating labor cost
Labor	Operating labor = US$200/h, other labor cost multiplier = 0.2	Labor cost = hourly rate × number of operators × number of equipment × number of lots/y	Fluorescence-activated cell sorting operator wage = £57,500/y Magnetic-activated cell sorting operator wage = £46,000/y	Salary by salary band, taking into account pension, overheads, and training costs	Operator cost = US$120,000/y
Facility costs	Depreciation over 10 years	Lang factor: 23.67 Maintenance (% capital investment) = 10% Depreciation (% capital investment) = 7%	N/A^a^	Office space, business rates, service charge cleanroom space costed per square meter Depreciation over 10 years	N/A^a^

^a^N/A: not applicable.

The cost assumptions of Harrison et al [25] were considerably more detailed than those in the rest of the articles. Quality control test panels were in accordance with good manufacturing practice requirements of the specific product and are listed in detail in the supplementary material of Harrison et al and based on industry information from Athersys. Depending on product characteristics, each product needs different quality control tests and assays requirements and, hence, the costs can be quite different. For instance, genetically modified cells would require assays on transformed cell populations to demonstrate appropriate and reproducible expression of newly acquired characteristics [25].

Labor costs can be quite different depending on geographical location. In an interview with the chief executive of Nanjing Legend, a Chinese company, he estimated that the manufacturing costs for CAR-T in China can be one-sixth of those in the United States due to cheaper overheads [32]. Simaria et al [22] suggested in their sensitivity analysis that labor rate is one of the most important cost drivers for less-automated processes.

The two main methods for accounting for facility costs in the studies were equipment-factored estimates (eg, Lang factor) and estimates of cost per square meter. Facility costs are averaged over the period of depreciation and shared among all doses. The Lang factor is a commonly used method in project cost estimates in the engineering industry and is recommended by the American Association of Cost Engineers [33]. The Lang factor used by Hassan et al [23] was taken from Pollock et al [12], which took into account pipework and installation, process control, instrumentation, electrical power, building works, detail engineering, construction and site management, commissioning, and contingency factor. It was unclear what Harrison et al [25] included in their method of cost per square meter applied to cleanroom space. In addition, a different cleanroom grade would constitute a different cleanroom space running cost and, hence, it is important to understand the good manufacturing practice requirements of the manufacturing environment.

### Solution Method

A solution method is required to relate the decision variables to the decision objective. Process models were built in all the identified studies. Table 6 [19-27] summarizes the approaches to solution methods used in the 9 articles. The two main approaches were process economics modeling in the form of what-if studies and multi-attribute decision making.

**Table 6:** Techniques and algorithms used for solution methods.

Technique or algorithm	Ungrin, 2012 [19]	McCall, 2013 [27]	Simaria, 2014 [22]	Hassan, 2015 [23]	Hassan, 2016 [21]	Lambrechts, 2016 [20]	Weil, 2017 [24]	Harrison, 2018 [25]	Jenkins, 2018 [26]
Process economics modeling	No	No	Yes	Yes	Yes	No	Yes	Yes	Yes
Value systems modeling	No	Yes	No	No	No	No	No	No	No
Design structure matrix	No	Yes	No	No	No	No	No	No	No
What-if scenario analysis	No	No	Yes	Yes	Yes	No	Yes	Yes	Yes
Multi-attribute decision making	No	No	No	No	No	No	No	No	Yes
Database evaluation	No	Yes	No	No	Yes	Yes	No	No	No
Latin hypercube	No	Yes	No	No	No	No	No	No	No
Monte Carlo simulation	No	No	No	No	Yes	No	No	No	Yes
Sensitivity analysis	No	No	Yes	No	No	No	Yes	No	Yes
Deterministic process evaluation	Yes	No	Yes	Yes	No	No	Yes	Yes	Yes
Stochastic model	No	Yes	No	No	Yes	No	No	No	Yes
Data Visualization	No	No	No	No	No	Yes	No	No	No

#### Process Economics and Value Systems Modeling

To ensure all relevant costs are identified, typically models simulating the actual manufacturing or product development process are constructed and costs associated with each step are summed. Costs were analyzed by Simaria et al [22], Hassan et al [23], Hassan et al [21], Weil et al [24], Harrison et al [25], and Jenkins and Farid [26]. This method allows for process-centric costing, which in turn supports cost analyses based on technology options.

A value system modeling is a way of modeling the firms by sets of activities that the firms use to create value and competitive advantage [34]. McCall [27] modeled the set of activities in product development and accounted for development process characteristics such as interdependency, iteration, activity cost, and duration uncertainties. Through this model, McCall was able to highlight the critical processes, resources, and risks in product development. The report highlighted the importance of early-stage investment, clinical trials rework, and regulatory requirements.

#### Design Structure Matrix

Design structure matrix is a method developed by Steward and other in 1981 for planning and communicating engineering works [35]. The matrix represents the events, their sequence, and the interdependencies between events. McCall [27] used a design structure matrix to clearly represent the precedence constraints while considering iteration circuits inherent in product development.

#### What-If Scenario Analysis

Several articles included what-if scenario analysis, where the dose sizes, lot sizes, and demand for products were varied. These studies were used to provide guidance for technology selection under different circumstances.

#### Single Objective Versus Multi-Attribute Decision Making

While most articles dealt only with manufacturing or investment cost optimization, Jenkins and Farid’s model employed a multicriteria decision-making methodology to assess bioprocess flowsheets [26].

The weighted sum technique provided a way to account for both quantitative and qualitative attributes of a solution, and, by assigning weightings, considered the perceived relative importance of different attributes. Weighted sum, however, is just one of many methods of multi-attribute decision making. Hester and Velasquez [36] conducted a comprehensive review and comparison of the methods commonly used. The analytic hierarchy process allows for pairwise comparisons to compare alternatives, which is less data intensive and more suitable for qualitative performance-type problems and resource management applications.

#### Handling of Risks and Uncertainties

Common themes incorporated into these manufacturing and development cost models are the risks and uncertainties lurking in the industry. The major methods of capturing risks and uncertainties in the studied models were stochastic modeling, Latin hypercube sampling, Monte Carlo analyses, and sensitivity analyses.

#### Deterministic Versus Stochastic Modeling

Deterministic models use discrete values, meaning that, for a certain input, the output will always be the same. Stochastic models have at least one quantity with random values, leading to an ensemble of different outputs [27].

Stochasticity was accounted for in 3 of the articles, where triangular distributions were applied to parameters to capture the uncertain and variable nature of the systems. McCall [27] applied a triangular probability distribution to the task duration to capture the uncertainties in development step duration. Hassan et al [21] applied a probability distribution to the success rate of each development step. Jenkins and Farid [26] assigned probability distributions to the weighting of quality attributes in the multi-attribute decision-making module; hence, they modeled only the variability in preference of quality attribute.

We noted that probability distribution had not been assigned to variability and uncertainties in the manufacturing process in these studies.

#### Latin Hypercube Sampling and Monte Carlo Simulation

McCall [27] categorized the risks into product risk factors and enterprise risk factors. Product risks were defined as risks that can harm the patient, namely the choice of cell type, manufacturing processes, and delivery mechanism. Enterprise risks were defined as risks that affect the commercialization of the product and the business developing the product, namely technical risks and market risks. The Latin hypercube sampling method was used to consider the probability of failure and duration for each task along the product development pathway and the interdependencies. It is worthwhile to note that in this model, iteration caused by failures and impact of failures during each phase were considered using three matrices: design structure, rework probability, and rework impact.

Hassan et al [21] simulated the risks and uncertainties of process change along the product development pathway through Monte Carlo analyses. To adjust the project NPV according to risk, they used a discount rate based on the riskiness and expected development time.

#### Sensitivity Analysis

Sensitivity analysis is a common strategy to account for uncertainties and identify key cost drivers [22,24,26]. It is useful in understanding which assumptions and parameters the overall system is most sensitive to. Simaria et al [22] focused on upstream production for MSCs and found that the main cost drivers were microcarrier area, harvest density, media price, and downstream yield. Weil et al [24] and Jenkins and Farid [26], who considered processes requiring differentiation or gene modification, consistently cited the key cost drivers to be the efficiency of differentiation and gene modification. This is particularly interesting, as Weil et al modeled an autologous process [24] while Jenkins and Farid modeled an allogeneic process with a different cell type [26].

#### Hypothetical Case Studies

Hypothetical case studies where the demand and dose sizes varied were conducted for manufacturing systems, including by Simaria et al [22], Hassan et al [23], Weil et al [24], and Jenkins and Farid [26]. The case studies were useful in the evaluation of process bottlenecks and technology-switch sweet spot analysis.

### Implementation

#### Model Validation

##### Data Mining

McCall [27] and Hassan et al [21] both established databases using real commercial case studies to inform some of their assumptions. McCall collected data from development programs surrounding orphan and non–orphan cell therapies [27], while Hassan et al collected information on clinical trial development times and failure rates of all 592 commercial cell therapy projects that entered development from 1981 to the end of 2011 [21]. These databases are useful for benchmarking purposes and increase the validity of the development time assumptions.

##### Laboratory Experiments

Ungrin et al [19], Weil et al [24], and Harrison et al [25] all included experimental results to inform assumptions in their studies. Using experimental results to support key assumptions is a powerful tool in validation. For instance, performance data of unit operations may not be as good as the vendor claims and the use case may be different, hence leading to varying results. Also, conducting experiments with different cell types can give valuable insights into characterization of the inherent variability of the process, lending the model more credibility.

#### Simulation Platforms

**Figure 5:**
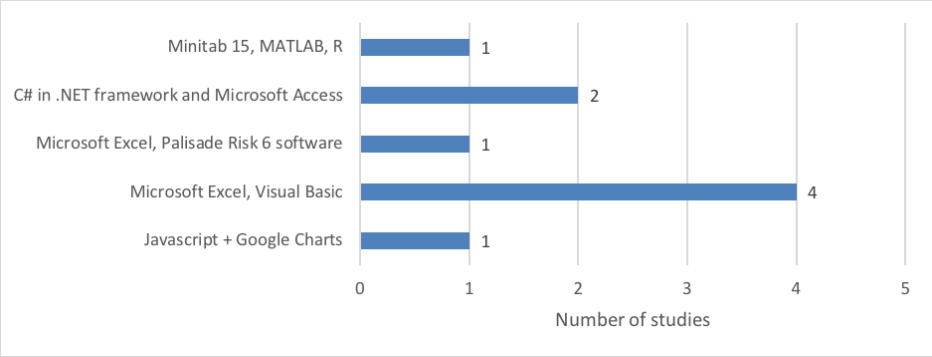
Choice of simulation platforms.

Figure 5 shows the simulation platforms employed in the different tools. Depending on the goal of the decisional tool, different simulation tools have been used. For simpler models, using Microsoft Excel with Visual Basic has appeared to be sufficient. For instance, the Excel model constructed by Weil et al [24] consisted of a cost model with mass balance, design, sizing, resource utilization and cost-of-goods equations, database of bioprocess technology data and iterative algorithms, and scenario analysis developed using Visual Basic. However, Visual Basic codes are susceptible to Excel program upgrades, and changing formats (eg, adding a column or a row) may cause changes in the functions. C# or MATLAB allows more versatile coding experience, and for models requiring many runs, such as uncertainty or stochasticity analysis, these platforms may be more suitable.

For models with larger databases, it is worth looking into database software such as Microsoft Access. Database software provides better scalability if the volume of data is huge, and the links can be built in a more robust ways than in spreadsheets.

Visualization software such as Google Charts allows for information to be easily updated and visualized and hence is useful for presenting a lot of data in a meaningful way [20]. Dedicated add-ons, such as Palisade Risk 6, used by McCall [27], allow for Monte Carlo simulations and sensitivity analysis to be carried out a bit more easily. Without experience of using the software, however, the usability of this software has not been formally compared with implementations based on C# or MATLAB.

## Discussion

### Principal Findings

We systematically reviewed 9 articles describing decisional tools in the regenerative medicine area. The diversity of decisions that have been modeled in this area is very limited compared with work relating to monoclonal antibodies and small-molecule pharmaceuticals. Some areas that are not yet studied include scheduling [37], facility fit [38], capacity planning [39,40], supply chain optimization [41,42], and portfolio management [6].

In terms of product development systems, it is worthwhile to consider that regenerative medicines for serious life-threatening diseases can be eligible for regulatory shortcuts through various early-access initiatives, such as breakthrough therapy designation in the United States [43], Priority Medicines (PRIME) in the European Union, and Sakigake Designation in Japan [43-47]. Table 7 [17,44,48-60] shows some examples of cell and gene therapy products that have been granted regulatory pathways or designations that allow for acceleration of the commercialization process. As more and more products are granted these designations, to assist decision making, the implications of these pathways should be considered. For instance, breakthrough therapy designation allows for New Drug Application and Biologic License Application data to be submitted as they are accumulated, and orphan drug designation allows for approval of medicinal products within 6 months. The impact of these accelerated regulatory pathways can be evaluated using decisional tools to better understand the efficiency of these policies and inform future regulatory framework improvements.

Available models provide good guidance for the industry in terms of technology evaluation at various scales for large-scale allogeneic process unit operations. However, autologous products make up a significant proportion of approved regenerative medicine products approved worldwide (19 out of 36) [61]. As of 2012, more than 65% of the stem cell clinical trials contained autologous cells or tissues [62] and therefore deserve attention in the future development of decisional tools. We also noted that, despite the widespread use of simulation in the existing decisional tools, none of these used optimization algorithms that can identify and select best candidate solutions.

Additionally, as Figure 3 shows, there are no tools modeling the entire needle-to-needle process. For a more comprehensive understanding to aid decision making, the whole process from patient to patient should be considered. For instance, tissue or cell procurement can be a major constraint on the lot size and final cell count. Population doubling levels should be considered, as a higher passage number has been shown to be negatively correlated to the therapeutic potential of the cultured MSCs [63]. Harrison et al [25] conducted experiments on 3 donor samples and through their model established the challenge of donor variability on equipment sizing and of expansion potential on the final cost of goods. To provide a comprehensive account of scheduling for administration of a therapy, the availability of hospital resources should be considered. Models can be extended to cover tissue procurement and institutional requirements surrounding therapy administration in order to optimize the cost and overall patient-to-patient supply chain robustness.

As noted previously, the efficiency of differentiation and gene modification steps have shown to be a key cost driver for both autologous and allogeneic cell therapies. More in-depth evaluation of different gene editing technologies may be beneficial for driving the industry to adopt more robust and cost-effective strategies in the process step.

Similar methodologies can be applied to other novel therapeutic modalities. The approvals of alipogene tiparvovec (Glybera) in the European Union in 2012 and of voretigene neparvovec (Luxturna) in the United States in 2017 show the potential of adeno-associated virus vector-based gene therapies [54,64]; over 50 clinical candidates are using adeno-associated virus vectors [65]. Furthermore, since the approval of the first clinical trial for the clusters of regularly interspaced short palindromic repeats (CRISPR) genome editing technology in 2016, there are now over 20 active trials registered on ClinicalTrials.gov.

**Table 7:** Examples of cell and gene therapy products that have been granted early-access designations.

Regulatory agency and regulatory pathway		Example cell and gene therapy products
**United States: Food and Drug Administration**		
	Priority review (1992)	Novartis: Kymriah
	Accelerated approval (1992)	Pfizer: bosutinib
	Fast track (1998)	Renova: RT-100 AC6 gene transfer (Ad5.hAC6); DNAtrix therapeutics: DNX-2401; AveXis: AVXS-101
	Breakthrough therapy (2012)	Enzyvant: RVT-802; Juno and Celgene: JCAR017; Adaptimmune and GlaxoSmithKline: NY-ESO-1c259T; Bluebird and Celgene: bb2121
	Expedited access pathway (2015)	Avita: Recell [48]
	Orphan drug designation (1983)	uniQure: AMT-130 [49]
	Rare pediatric disease priority review (2014)	Spark Therapeutics: Luxturna [17]
	Regenerative medicine advanced therapy designation (2017)	Abeona Therapeutics: ABO-102 [50]; Mesoblast: mesenchymal precursor cell therapy [51]
**European Union: European Medicines Agency**		
	Accelerated assessment (2004) [52]	Bluebird: LentiGlobin [53]
	Orphan drug designation (2000) [52]	uniQure: AMT-130; Orchard Therapeutics: Strimvelis
	Marketing authorization under exceptional circumstances (2005) [52]	uniQure: Glybera [54]
	Conditional marketing authorization (2006) [52]	Chiesi Farmaceutici: Holoclar [44]; MolMed: Zalmoxis [55]
	Adaptive pathway (2015) [52]	Atara Bio: ATA129
	Priority Medicines (PRIME) (2016) [52]	uniQure: AMT-060, AMT-061; Juno and Celgene: JCAR017; Bluebird: LentiGlobin [53]; Adaptimmune and GlaxoSmithKline: NY-ESO-1c259T; Bluebird and Celgene: bb2121 [56]
**Japan: Pharmaceuticals and Medical Devices Agency**		
	Priority review [57]	Glecaprevir/Pibrentasvir, AbbVie
	Orphan designation (1993) [57]	Edison Pharmaceuticals: EPI-743
	Conditional and time-limited approval (2014) [57]	No examples available
	Sakigake forerunner review assignment (2015) [58]	Nippon-Shinyaku: NS-065/NCNP-01 [59]
**China: State Administration for Market Regulation**		
	Accelerated and conditional approval (draft issued in 2017) [60]	Not yet in practice

As the industry moves toward delivering these novel therapies, learning from past clinical translation experiences (eg, the monoclonal antibody industry), better understanding of the risks, and making better informed decisions become all the more important.

### Strengths and Limitations

There were some limitations to the review process. First, we focused our search on the MEDLINE database because preliminary scoping searches suggested that there would be more targeted literature in these databases than in those available in EMBASE and Scopus. This decision increased the risk of not identifying all relevant articles. Second, due to time limitations, we did not engage a second reviewer to review articles for eligibility, increasing the risk of excluding eligible reviews due to oversight. We consulted the Cochrane Library and PROSPERO database retrospectively, and we found no reviews to be relevant to the review question.

These limitations notwithstanding, this study is, to our knowledge, the first to systematically review the methods and logic for the design of decisional tools in aiding regenerative medicine translation and manufacturing. The small number of published studies highlights the opportunities to develop further decision support tools for different decisions and product types. The detailed design method analysis can be helpful for future development of these tools in a systematic manner in order to facilitate the translation of novel therapies into clinics more time and cost efficiently. Furthermore, the identification of the gaps in the literature can be useful for other bioprocess researchers working in the area.

### Conclusions

We systematically reviewed the decisional tool landscape for regenerative medicine. Decisional tools have been shown to be instrumental in the commercialization of monoclonal antibodies through informing various decisions in manufacturing technologies, capacity planning, scheduling, and investment. As more and more regenerative medicine products receive regulatory approval, decisional tools offer a systematic way of evaluating different commercialization decisions and options. Studies within the regenerative medicine area have largely addressed the manufacturing challenges and cost-reduction drivers for allogeneic cell therapies. Decisional tools in tissue engineering and gene therapies are lacking. To more comprehensively understand the overall costs and supply chain robustness of these lifesaving cell therapies, the entire process from tissue procurement to postadministration should be considered. To put forward industrially relevant decisional tools, costs and process assumptions should be industrially validated to ensure that any results derived from the model are useful and relevant. Future decisional tools to integrate the different facets of the available decisional tools should be developed to inform decision making in the rapidly expanding and transformative field of regenerative medicine.

## Appendix

Multimedia Appendix 1 Executed data abstraction form.

## Abbreviations

CAR-T: chimeric antigen receptor T cells
FACS: fluorescence-activated cell sorting
MACS: magnetic-activated cell sorting
MSC: mesenchymal stem cell
NPV: net present value
PRIME: Priority Medicines
PRISMA: Preferred Reporting Items for Systematic Reviews and Meta-Analyses
