# Supplementary material for: Decision Support Tools for Regenerative Medicine: Systematic Review
Source: J Med Internet Res. 2018 Dec 19;20(12):e12448. doi: 10.2196/12448 (PMC6315273; doi:10.2196/12448)
Supplement: Multimedia Appendix 1 [file jmir_v20i12e12448_app1.pdf]

Executed data abstraction form

Title	Decision objective	System		Decision-making	Implementation	
	Type of decision	System boundary	Product type	Techniques or algorithms	Model application and validation	Simulation platform
**Ungrin 2012**	Operational yield for cell expansion process	Bioprocess: cell culture	hPSC, endoderm progenitors	Monitoring yield loss parameter by varying input process parameters	Laboratory experiments to investigate cellular dynamics and mechanisms of differentiation processes	Minitab 15, MATLAB, R
**McCall 2013**	Investment costs	Product development: preclinical trials to phase 3 clinical trials	N/A	Design structure matrix; Latin hypercube sampling methods; discrete event simulation; data mining	Case study comparing new drug development via non-orphan and orphan drug pathways for Acute Ayocardial infarction	Microsoft Excel, Visual Basic
**Simaria et al 2014**	Upstream manufacturing cost of goods	Manufacturing: cell culture	Allogeneic cells	Deterministic process evaluation algorithm; economic and technical components; what-if scenario; database evaluation	Hypothetical case study	C# in .NET framework and Microsoft Access
**Hassan 2015**	Downstream manufacturing cost of goods	Manufacturing: wash and concentration	Allogeneic MSCs	Deterministic process evaluation algorithm; what-if scenario; economic and technical components; database evaluation	Hypothetical case study	C# in .NET framework and Microsoft Access

Title	Decision variables						
	USP	DSP	Scale and throughput	Model input parameters	Model output	Model assumptions	Constraints (if identified)
**Ungrin 2012**	Expansion and differentiation	N/A	10^9 cells/dose	- Number of live cells counted<br>- Cumulative number of population doublings	- Yield loss parameter	- physiochemical limitations define upper limit of aggregate size	Not described
**McCall 2013**	N/A	N/A	N/A	-Duration of task (used to model uncertainty and complexity within the domain of the task)<br>- Learning from iteration	cumulative cost of completion of all tasks at the end of the simulation run:<br>- Sum of all the products of individual task duration and cash-burn level	- Fixed, renewable resource pool throughout the entire project duration<br>- Constant resource requirement over the entire period for each task	Resource constraint (fixed, renewable resource pool)
**Simaria et al 2014**	Expansion: T-flask vs Multi-layers vs compact flasks vs compact multi-layers vs hollow fibre bioreactors	N/A	Allogeneic,<br>Lot size: 50-1000 doses/lot<br>Demand: 1000-500,000 doses per year	Product:<br>- Dose<br>Scale:<br>- Demand<br>- Lot size<br>Process:<br>- Seeding & harvest density<br>- Nr expansion stages<br>- Global process yield<br>Candidate technologies:<br>- Growth surface area<br>- Price<br>- Labour, media, equipment requirements	Optimal cell expansion strategy for each manufacturing scale<br>- Type of technology and nr units to be used at each expansion stage<br>- COG/dose<br>- COG breakdown: materials, labour, QC and eq depreciation costs	cost assumptions<br>Process parameters assumptions	Manufacturing constraints: maximum number of cell expansion technology units handled per lot in the last expansion stage
**Hassan 2015**	N/A	Wash and concentration step: Tangential flow filtration vs fluidised bed centrifugation	Allogeneic,<br>- final cells/ml: 0.3M to 10M<br>- lot size: 50 to 10,000 doses/lot<br>- demand: 1000 -	- Number of cells and total volume post-expansion and trypsinization	DSP COG/dose<br>- Annual direct operating costs and indirect costs: materials, labour, QC, facility-dependent depreciation and maintenance costs); divided	cost assumptions<br>Process parameters assumptions<br>Microcarrier use upstream	- Fixed upstream strategy<br>- Limit of one-equipment unit for volume reduction and washing per lot<br>- Tighter or more relaxed time constraints for different cell types<br>- target final concentration at the end of the flowsheet

	Filling and cryopreservation: Cryovial filling machine vs cryovial freezer	500,000 doses/year		by<br>- Output in number of doses/year		taking into account minimum harvest volumes for volume reduction technologies

Title	Decision objective	System		Decision-making	Implementation	
	Type of decision	System boundary	Product type	Techniques or algorithms	Model application and validation	Simulation platform
**Hassan 2016**	Investment costs, Project net present value	Product development: Phase 1 clinical trials to regulatory approval Manufacturing: cell culture	Allogeneic MSCs	Deterministic process evaluation algorithm, economic and technical components; Monte Carlo analysis; database with information on clinical trial development times and failure rates of cell therapy project from 1981 to 2011	Hypothetical case study	Microsoft Excel and Palisade Risk 6 software
**Lamb-rechts 2016**	N/A	Bioprocess: cell culture	MSC/Stromal cells	Database of individual cell expansion processes, visualization tool, cost comparison	Data from published studies	JavaScript, Google Charts
**Weil 2017**	Manufacturing cost of goods	Manufacturing: Purification	iPSC-derived progenitor photoreceptors	Laboratory experimental results input into bioprocess evaluation algorithm; what-if scenario; economic and technical components; database evaluation	Case study comparing different DSP affinity purification strategies for production of photoreceptor progenitor cells with experimental data	Microsoft Excel, Visual Basic
**Harrison 2018**	Manufacturing cost of goods	Manufacturing: cell culture to cryopreservation	MSC	Process economics modeling with economic and technical components; what-if scenario	Facility modeled on a real manufacturing facility in UK with real experimental data	Microsoft Excel, Visual Basic
**Jenkins 2018**	Manufacturing cost of goods	Manufacturing: cell culture to purification	Allogeneic CAR-T	Process economics modeling with economic and technical components; what-if scenario; database evaluation; Multi-attribute decision-making	Hypothetical case study	Microsoft Excel, Visual Basic

Title	Decision variables						
	USP	DSP	Scale and throughput	Model input parameters	Model output	Model assumptions	Constraints (if identified)
**Hassan 2016**	Expansion: T-flasks vs microca	N/A	Allogeneic, - 2 x 10^8 cells/dose - demand: 10,000-	- Parameters relating to the development stage of the project	- Risk-adjusted net present value	Assumptions of activities involved in the	Not described

	rriers with single use bioreactors		100,000 patients/ year	- Allogeneic/ autologous<br>- Dosage per treatment<br>- Clinical application<br>- Planning process technologies<br>- Proposed product price<br>- Expected market uptake<br>- Cost of capital<br>- Tax rate<br>- Staff requirements	- Payback time<br>- Reimbursement<br>- Expected cost of development<br>- Expected out-of-pocket cost per phase	development lifecycle when it occurs, cost basis and potential impact	
**Lamb-rechts 2016**	Expansion	N/A	Scale of processes with final cell numbers ranged between 7.5 x 10^6 and 1.1x10^10	- Type of culture vessel<br>- Expansion factor<br>- Final cell yield<br>- Medium composition<br>- Population doubling time<br>- Growth surface and coating	Visualization model	Comparable quality of data from published papers	Only papers with sufficient information were included in the study.<br>Papers from university setting, non GMP setting
**Weil 2017**	N/A	Affinity purification: FACS vs MACS vs novel beads (SpheriTech)	Dose sizes ranging from 2 x 10^7 to 2 x 10^8	Purification process parameters:<br>- Purification method<br>- Number of units required to process given cell population	Whole bioprocess COG/dose<br>- Detailed COG breakdown: Material, labour, Equipment depreciation, tax, insurance, maintenance (using a factor described in Jenkins 2016)	cost assumptions Process parameters assumptions	- Purification yield<br>- Purity<br>- Throughput<br>- Turnaround (preparation and cleaning)<br>- Differentiation efficiency
**Harrison 2018**	Expansion: monolayer expansion platforms	Transport: cryogenic vs fresh	Allogeneic, 2500 doses/year per regional manufacturing center	- Total patient doses required per year<br>- Biological characterisation of cell sources	- lot size yield projections<br>- resource consumption<br>-	Process and cost assumptions, facility size, office space, staffing requirements	Not described

	manual vs automated processes, serum-containing media vs serum-free media				equipment requirements<br>- COG consisting of annual direct costs and indirect costs		
**Jenkins 2018**	Expansion: Rocking motion bioreactor, planar culture flask, gas permeable vessel, integrated bioprocess platform	Concentration: Fluidised bed centrifugation, spinning filter membrane, integrated bioprocess platform<br>Purification: MACS, integrated bioprocess platform	Allogeneic,<br>- Dose sizes: 10^7 to 10^9<br>- demand: 500- 5000 doses per year	- Annual demand<br>- dose size<br>- Process parameters<br>- Weightings assigned to each attribute<br>- Ratings assigned to each attribute for each process strategy tested using the analysis<br>- probability distribution used as inputs for Monte Carlo simulation	- Whole bioprocess COG/dose<br>- Culture vessels required per lot<br>- Lots required at certain demand	weighting and ratings of financial and operational attributes, process and cost assumptions	- Capacity constraints of units
